# Synthesis and evaluation of resveratrol–cerium modified hydroxyapatite for enhanced bone repair scaffolds

**DOI:** 10.1039/d5ra10060g

**Published:** 2026-02-23

**Authors:** Dezhou Wang, Min Guo, Yuqi Gao, Shengrui Gao, Wanzhong Yin, Wenzhi Song

**Affiliations:** a Stomatology Department, China–Japan Union Hospital of Jilin University Changchun 130033 PR China songwz@jlu.edu.cn; b Laboratory of Polymer Materials Engineering, Changchun Institute of Applied Chemistry, Chinese Academy of Sciences Changchun 130022 PR China; c Department of Otorhinolaryngology, The First Hospital of Jilin University 1 Xinmin Street Changchun 130021 PR China yinwz@jlu.edu.cn

## Abstract

Metal-phenolic networks (MPNs) are a category of amorphous coordination network materials formed by metal ions and phenolic ligands. They can be integrated into matrix composites to significantly enhance the overall functionality of the composites. In this study, to leverage the anti-inflammatory, antibacterial, and osteogenic properties of resveratrol (Res) and cerium (Ce), an innovative Res–Ce MPN was synthesized to modify hydroxyapatite (HA) nanoparticles. These Res–Ce/HA nanoparticles were then blended with polycaprolactone (PCL) to fabricate 3D-printed bone repair scaffolds. The results showed that the composite scaffold containing 10% Res–Ce/HA nanoparticles (PCL@10Res–Ce/HA) exhibited improved antibacterial activity. *In vitro* experiments revealed that Res–Ce MPNs in the PCL@10Res–Ce/HA scaffold notably enhanced the adhesion and proliferation of MC3T3-E1 cells on the scaffold surface. Simultaneously, they upregulated the expression of Runx2 and BMP2, and thus facilitated the osteogenic differentiation of cells. Furthermore, *in vivo* rat tibial defect repair experiments demonstrated that the 3D-printed PCL@10Res–Ce/HA scaffold remarkably promoted osteogenesis by upregulating BMP2 expression. Additionally, Res–Ce MPNs in the PCL@10Res–Ce/HA scaffold inhibited excessive inflammation, thereby supporting bone regeneration. Importantly, comprehensive biosafety evaluations confirmed the clinical feasibility of the PCL@10Res–Ce/HA scaffold. Collectively, these findings indicate that the PCL@Res–Ce/HA scaffold with optimized composition integrates anti-inflammatory, immunomodulatory, and bone defect repair capabilities, making it a promising candidate material for bone defect repair.

## Abstract 2

PCL scaffolds incorporated with Res–Ce MPN-modified HA nanoparticles were prepared. They show synergistic antibacterial, anti-inflammatory, and osteogenic activities, effectively promoting bone defect repair with excellent biosafety.

## Graphical abstract

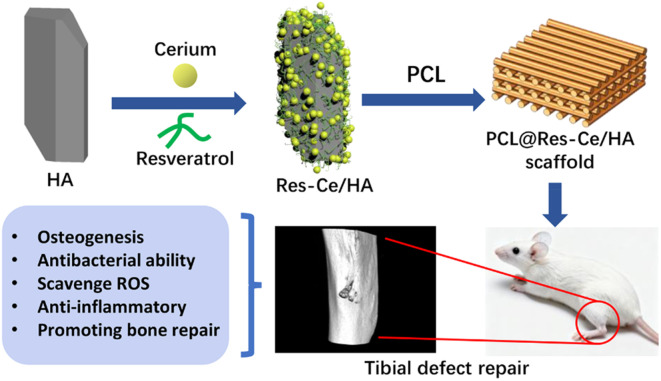

## 1 Introduction

Large bone defect repair remains a major challenge in the field of bone regeneration. Currently, autologous and allogeneic transplantation stand as the most prevalent approaches. Nevertheless, these methods are plagued by certain drawbacks that hinder their clinical application, such as limited donor sources, risks of immune rejection, and susceptibility to infection.^1–3^ In contrast, the application of tissue-engineered scaffolds offers the potential to address the shortage of bone grafts. Among these, 3D-printed scaffolds are capable of providing adequate mechanical strength and a porous structure, both of which are conducive to offering mechanical support and facilitating the exchange of nutrients and metabolites. Moreover, they enable personalized treatment strategies tailored to the variability of clinical cases.^4,5^

Polycaprolactone (PCL) is often regarded as an ideal substrate for 3D-printed bone scaffolds due to its excellent machinability, biocompatibility and degradability.^6^ However, in order to address the inadequacy of PCL scaffolds in osteogenic activity, inorganic materials like nano-hydroxyapatite (nHA) are frequently incorporated.^7,8^ Recent studies are increasingly focusing on biological functions beyond osteogenesis—such as regulating inflammation, antioxidant activity, and anti-infective capacity—all of which exert a substantial influence on bone repair. The integration of active agents, various metal oxides, and rare earth elements with nHA has emerged as an effective approach to enriching the biological functions of materials and further enhancing the bone repair efficacy of scaffolds.^9–11^ Innovative modifications to nHA have also emerged as a new research hotspot.

Polyphenols are ubiquitous natural compounds consisting of two or more phenolic structural units. As a class of economically feasible, environmentally benign, and biocompatible candidate compounds, polyphenols have garnered extensive research interest owing to their distinctive physicochemical properties.^12^ Resveratrol (Res) is a naturally occurring polyphenolic compound present in a variety of plants, including grapes, peanuts, and berries. It offers a wide range of health-promoting properties: antioxidant,^13^ anti-inflammatory,^14^ cardioprotective,^15^ antitumor,^16^ and antidiabetic effects.^17^ Additionally, it exhibits significant antibacterial activity against various pathogens.^18^ Numerous *in vitro* and *in vivo* studies have underscored bone–protective properties of Res, highlighting its role as a stimulator of osteoblast proliferation and an antagonist of osteoclast differentiation.^19^ Recent studies on the mechanism of Res in bone repair have been gradually lucubrated. Res can alleviate the inhibition of osteogenic differentiation of bone marrow mesenchymal stem cells induced by tumor necrosis factor-α (TNF-α), thereby delaying the progression of osteoporosis. The enhancement of the Wnt signaling pathway, activation of sirtuin 1 (Sirt1), and acetylation of runt-related transcription factor 2 (Runx2) have been identified as potential mechanisms through which Res exerts these effects.^20^ Furthermore, Res was also proved to activate the Src kinase-dependent estrogen receptor (ER) to stimulate osteoblasts to produce bone morphogenetic protein 2 (BMP2), while also increasing the serum concentration of BMP-2 *in vivo*.^21^ On the other hand, Res positively regulates the expression and activity of Piezo1 by activating Sirt1, thereby activating the Piezo1 channel and triggering calcium ion influx. As a mechanosensitive channel protein, Piezo1 plays a crucial role in promoting bone formation and repair.^22,23^ In addition, Res can significantly enhance the immunomodulatory capacity of stem cells. In periodontitis animal models, it could partially mitigate bone loss by activating endogenous somatic stem cells and inhibiting the infiltration of inflammatory T cells.^24^ However, Res exhibits poor water solubility under physiological pH conditions and undergoes rapid degradation, leading to extremely low systemic bioavailability and thus severely impeding its clinical translation.^25^

Metal-phenolic networks (MPNs) are a class of amorphous coordination network materials composed of metal ions and phenolic ligands. Owing to their abundant phenolic hydroxyl groups, MPNs can efficiently interact with various functional components through multiple mechanisms, including hydrogen bonding, coordination, π–π stacking, and hydrophobic interactions. Furthermore, the intrinsic properties of MPNs, such as high porosity, pH responsiveness, and metal selectivity, can be incorporated into MPN-based composites, markedly enhancing their overall functionality.^26^ By integrating functional components (*e.g.*, drug molecules, proteins, and nanoparticles), diverse functional MPN composites can be fabricated. These materials have found widespread applications in fields such as separation science, energy storage, drug delivery, catalysis, and bioimaging.^27^ Several studies have reported the use of Res as a phenolic compound to fabricate MPNs, aiming to improve the drug's utilization efficiency. In Jia's report, the Res–magnesium MPN was fabricated and integrated with an innovative photosensitive poly-l-lysine gel. This design allows for the enables sustained release of Res and synergistically enhances the expression of VEGF and also promotes resistance to tensile forces, aided by Mg ions, in an anastomotic tracheal fistula animal model. Moreover, the Res effectively inhibits bacteria, reduces local expression of key inflammatory factors, and induces polarization of macrophages toward an anti-inflammatory phenotype, as well as inhibits TGF-beta 1, consequently decreasing collagen production levels in an animal model of post-tracheal resection.^28^

Cerium (Ce) is the most abundant element in the lanthanide series of rare earth elements. It exhibits both metallic reactivity and chemical stability, and occurs in the Ce^4+^ and Ce^3+^ oxidation states under normal conditions. Ce possesses multiple electron orbits. A favorable redox potential exists between Ce^4+^ and Ce^3+^. And its electronic structure confers excellent redox catalytic properties.^29^ Ce ions exhibit pH-dependent enzyme–mimetic activity under acidic extracellular infection conditions. Specifically, they display peroxidase/oxidase (POD/OXD)-like activity to generate reactive oxygen species (ROS) in acidic environments associated with infection, while exerting superoxide dismutase/catalase (SOD/CAT)-like activity to scavenge excessive ROS under neutral or alkaline conditions. Due to its outstanding nanozyme activity, Ce has already been used to assemble MPNs. Zeng *et al.* explored a shell–core structured biomaterial, consisting of a gelatin nanoparticle (GNP) platform loaded with bone morphogenetic protein 9 (BMP9) and coated with a tannin (TA)–Ce MPN. Extracellularly, it rapidly responded to lower pH, achieving specific release in an inflammatory microenvironment. Intracellularly, it impacted the formation, function, and differentiation of osteoclasts through the macrophage–osteoclast axis, thereby promoting bone defect repair.^30^ In Song's study, the macrophage cell membrane-camouflaged nanoparticle was prepared. It was a kind of MPN assembled from epigallocatechin gallate (EGCG)and Ce^4+^. Owing to its geometric similarity to the active metal sites of natural antioxidant enzymes, EC exhibits high scavenging efficiency toward various ROS and reactive nitrogen species (RNS). Meanwhile, the macrophage cell membrane facilitates M-EC in evading the immune system, being internalized by inflammatory cells, and specifically binding to interleukin-1β (IL-1β).^31^ All the previous studies have laid the groundwork for the design of a novel osteogenic material. The nHA modified with Res–Ce MPN could be constructed. This combination integrates anti-inflammatory, immunomodulatory and bone defect repair biological functions, making it an ideal material for the repair of bone defects.

**Scheme 1:**
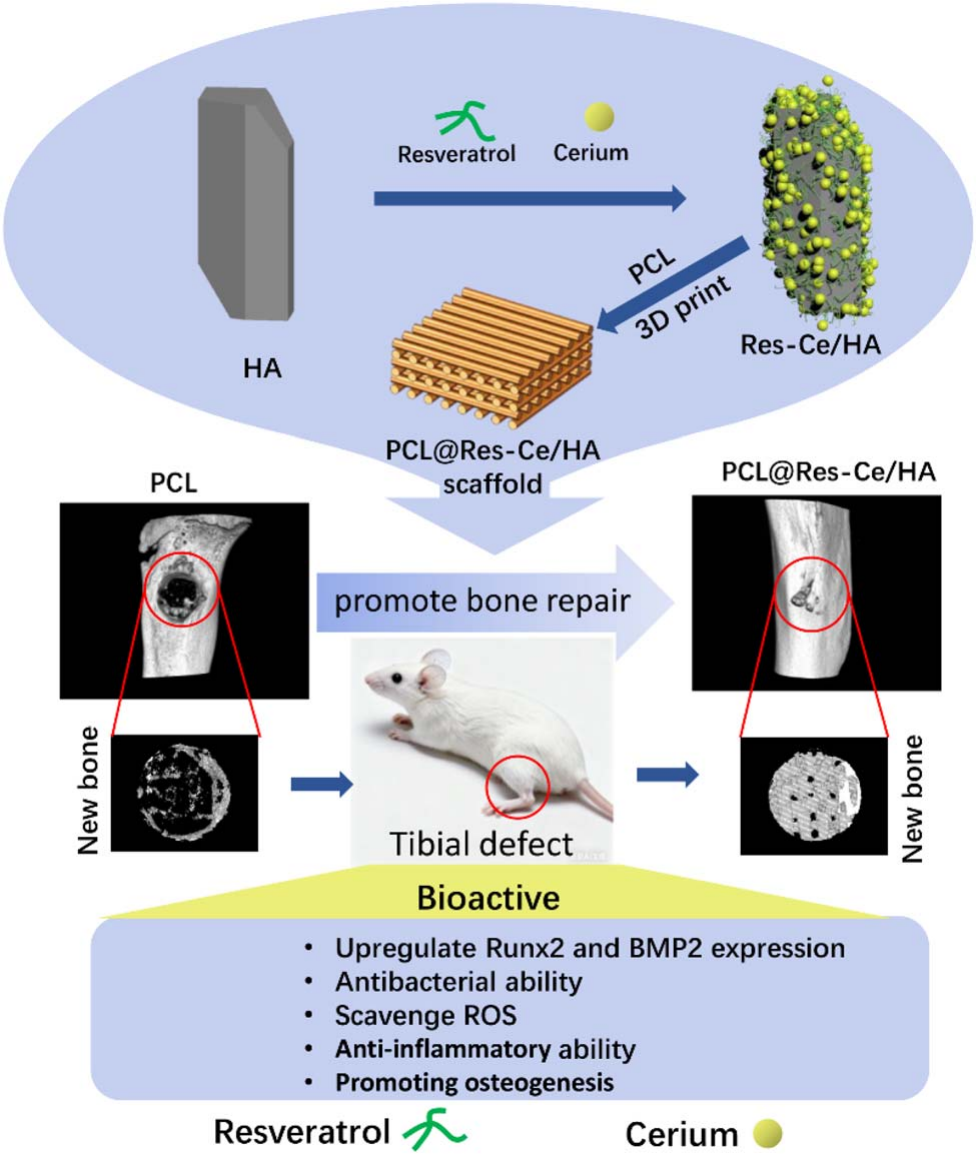
Schematic diagram showing the multifunctional mechanisms of Res–Ce MPNs in enhancing antibacterial, anti-inflammatory and osteogenic capacities of PCL@Res–Ce/HA scaffolds for bone defect repair.

In the present research, specifically, by combining Res with Ce, a novel MPN system was constructed, which was further used to modify nano-hydroxyapatite (Res–Ce/HA). Subsequently, different amount of Res–Ce/HA was incorporated into the PCL to fabricate a series of bone scaffolds *via* 3D printing. The physicochemical properties and biological activities of various scaffolds were evaluated to optimize the formulation of the composites. Meanwhile, the anti-oxidant, antibacterial property, and osteogenesis induction of the composite scaffold were evaluated *in vitro*. At last, the bone repair capacity and biosafety of the composite scaffold were assessed using a rat tibial defect repair model (Scheme 1).

## 2 Experimental section

### 2.1 Materials

Res and cerium sulfate (Ce(SO_4_)_2_) were purchased from Shanghai Aladdin Biochemical Technology Co., Ltd (China); PCL (average molecular weight = 100 000 Da) was purchased from Jilin Sinotech Healthy Technology Co., Ltd (China). Unless otherwise specified, all the reagents were purchased from Sigma-Aldrich. The MC3T3-E1 cell line was purchased from the Cell Bank of the Chinese Academy of Sciences.

### 2.2 Synthesis and characterization of Res–Ce/HA

The nHA was synthesized by the hydrothermal method as previously reported.^32^ 2 g of nHA was added to 200 mL of 10% ethanol aqueous solution, followed by the addition of 120 mL of a 6.4 g per L Res ethanol solution. The mixture was sonicated for 30 minutes to ensure uniform dispersion of nHA in liquid. Subsequently, under magnetic stirring (200 rpm), 100 mL of a 9.7 g per L Ce(SO_4_)_2_ aqueous solution was added to the suspension obtained in the previous step. After that, 1 M sodium hydroxide solution was slowly injected to adjust the pH value of suspension to 8.0. The reaction lasted for 1 h. After the end of reaction, the precipitated nanoparticles were collected by centrifugation at 8000 rpm and then thoroughly washed with ddH_2_O for three times. The Res–Ce/HA nanoparticles were finally obtained after a freeze-drying process.

The microscopic crystal structure and size of Res–Ce/HA nanoparticle was characterized by transmission electron microscope (TEM, FEI Tecnai G2 S-Twin). Scanning electron microscopy (SEM, ZEISS Gemini 2, Germany) with mapping scan was used to observe the micromorphology and key element (Ca & Ce) distribution of the nanoparticles. Fourier transform infrared (FT-IR) spectroscopic analysis was carried out using a Bio-Rad Win-IR spectrophotometer (Watford, UK) by using the potassium bromide (KBr) slice method to analyze the chemical composition of the Res–Ce/HA nanoparticles. The X-ray diffraction (XRD) data from 10° to 80° (2*θ*) were obtained to confirm the crystal structure of the Res–Ce/HA nanoparticles using a D8 Advance diffractometer (Bruker Co., Germany). X-ray photoelectron spectroscopy (XPS, Thermo) was proceeded to detect the elemental compositions. The amounts of Res capped on nanoparticles' surfaces were measured by thermogravimetric analysis (TGA, TA Instruments TGA500, USA). The samples were heated from 25 °C to 800 °C at a rate of 10 °C min^−1^ under air atmosphere.

### 2.3 Fabrication and characterization of 3D-printed scaffolds

Firstly, the Res–Ce/HA nanoparticles were mixed with PCL at different mass percentages (2.5 wt%, 5 wt%, 7.5 wt%, and 10 wt%) in a Banbury mixer, followed by dividing the composite into particles with a size of 5 mm × 3 mm for 3D printing. A biological Fused Deposition Modeling (FDM) 3D printer (Ubbiotech Co., Ltd) was used to fabricate scaffolds. The printing model was designed into billet of 20 mm × 20 mm × 2 mm with the pore size of 400 µm. The 3D printing parameters were set as extrusion rate 135%, printing speed 10 mm s^−1^, the base platform temperature was 15 °C for solidification of scaffolds. As control, both the PCL 3D-printed scaffolds without and with 10% nHA were prepared. The contents of each component in different scaffolds are listed in Table 1.

**Table 1:** The contents of each component in different groups

Groups	PCL (wt%)	HA (wt%)	Res–Ce/HA (wt%)
PCL	100.0	N/A	N/A
PCL@10HA	90.0	10.0	N/A
PCL@2.5Res–Ce/HA	97.5	N/A	2.5
PCL@5Res–Ce/HA	95.0	N/A	5.0
PCL@7.5Res–Ce/HA	92.5	N/A	7.5
PCL@10Res–Ce/HA	90.0	N/A	10.0

The samples from different groups (PCL, PCL@10HA, PCL@2.5Res–Ce/HA, PCL@5Res–Ce/HA, PCL@7.5Res–Ce/HA, and PCL@10Res–Ce/HA) were observed by a SEM (Gemini 2, Zeiss). And the element maps of the scaffolds' brittle fracture surfaces were analyzed by energy dispersive spectrometer (EDS, Gemini 2, Zeiss). The compression and tensile strength of scaffolds were test by a universal testing machine (Instron 5982, INSTRON, US), with the tensile and compressive speeds set at 5 mm s^−1^ and the ambient temperature maintained at 25 °C. The samples for mechanical test were designed and fabricated into the sizes shown in Fig. S1. The thermal weight loss of each sample was measured by TGA (TA Instruments TGA500, USA). The samples were heated from 25 °C to 800 °C at a rate of 10 °C min^−1^ under air atmosphere. The chemical structure of samples was analyzed by FT-IR (PerkinElmer, FT-IR-2000). The scaffolds were immersed in lipase solution (0.4 mg mL^−1^) and degraded at 37 °C. After 21 days of degradation, the weight loss (%) was calculated using the following formula:Weight loss rate (%) = *W*_0_ − *W*_t_/*W*_0_ × 100%*W*_t_ represents the scaffold weight at 21 days, and *W*_0_ is the weight of the scaffold before degradation.

### 2.4 Antibacterial assay

10 mL of *Escherichia coli* (*E. coli*, Gram-negative bacteria) or *Staphylococcus aureus* (*S. aureus*, Gram-positive bacteria) (1 × 10^4^ CFU mL^−1^) was incubated with 100 mg of different scaffolds respectively, at 37 °C with shaking of 150 rpm for 1 hour. Then bacteria were centrifuged at 4000*g* for 10 min, and then the supernatant was removed. The bacteria were resuspended and serially diluted 1 × 10^3^-fold with PBS. A 100 µL portion of the diluted bacteria solution was spread on the solid LB agar plate, and the colonies formed after 12 h incubation at 37 °C. The number of colony-forming units (CFU) was counted. The group of PCL was set as control group. The antibacterial rate was calculated using the following formula:Antibacterial rate (%) = CFU_treat_/CFU_control_ × 100%

Bacterial suspensions were separately plated onto LB agar plates, followed by placement of different group scaffolds at the center of each plate. The plates were incubated at 37 °C for 12 h, after which images were captured to examine the formation of inhibition zones. Solutions containing Res–Ce/HA nanoparticles at various concentrations (ranging from 0.2 mg mL^−1^ to 2 mg mL^−1^) were prepared using *E. coli* and *S. aureus* LB bacterial suspensions with an initial concentration of 1 × 10^5^ CFU mL^−1^. The mixtures were then incubated with shaking at 37 °C for 12 h, after which the turbidity of each well was observed. The minimum concentration of nanoparticles at which no bacterial growth was detected was defined as the minimum inhibitory concentration (MIC).

### 2.5 Morphology and proliferation of MC3T3-E1 cells on the scaffolds

MC3T3-E1 cells were cultured and expanded in high-glucose Dulbecco's Modified Eagle Medium (HG-DMEM, Servicebio) containing 10% fetal bovine serum (FBS, Gibco), 100 mg per L streptomycin and 63 mg per L penicillin, maintained under standard conditions of 37 °C and 5% CO_2_. Cells at passage 2 (P2) were detached *via* enzymatic digestion with 0.25% trypsin–EDTA solution, then seeded onto various scaffolds at a density of 1.5 × 10^4^ cells per well. The culture medium was refreshed every 48 hours. On day 3, the cells on the scaffolds were stained with the calcein-AM/PI live/dead cell staining kit (Beyotime, China), followed by imaging with an epifluorescence microscope (Zeiss, Imager Z2, Germany). The cells were fixed with 2.5% glutaraldehyde for 15 min, followed by dehydration with an ethanol gradient of 50% to 100%. Finally, the dried scaffolds were subjected to SEM imaging (SEM, ZEISS Gemini 2, Germany). Cell proliferation was assessed at day 3 and 5 using CCK-8 assay (Beyotime). At each timepoint, culture medium was replaced with serum-free medium containing 10% CCK-8 reagent (v/v) for 2 h incubation under standard conditions of 37 °C and 5% CO_2_. Subsequently, 200 µL of supernatant were transferred to 96-well plates for optical density measurement at 450 nm using a microplate reader (Thermo Fisher Scientific, USA). Cell counting analysis of the DAPI-stained images of cells adhered to the scaffolds were performed with ImageJ software (NIH, US).

### 2.6 Osteogenic activity of MC3T3-E1 cells on the scaffolds

The osteogenic differentiation of MC3T3-E1 cells grown on scaffolds was assessed by detecting alkaline phosphatase (ALP) activity after a 7-day culture.^33^ For quantitative detection, cells were rinsed with ice-cold PBS and lysed in RIPA buffer containing phenylmethanesulfonyl fluoride (PMSF, 1 : 100 v/v), with three subsequent freeze–thaw cycles (alternating between −80 °C and 25 °C). The lysate was centrifuged at 12 000*g* for 10 minutes at 4 °C to obtain clear supernatants, which were then used for enzymatic activity quantification. This was performed using *p*-nitrophenyl phosphate (pNPP) substrate (Solarbio, China), with absorbance measured kinetically at 405 nm. Calcium deposition in MC3T3-E1 cells at 14 days of culture was examined using the alizarin red S (ARS) assay.^34^ Cells were fixed with 4% paraformaldehyde at 4 °C for 30 minutes, after which ARS staining was performed following a standardized procedure. Scaffolds were incubated with 1% ARS solution (pH 4.2, Solarbio) under dark and humidified conditions (37 °C, 30 minutes) to enable specific binding of ARS to calcium–phosphate complexes. For quantitative analysis *via* spectrophotometry, ARS-calcium complexes were then dissolved by orbital shaking (150 rpm) in 10% cetylpyridinium chloride (CPC, Aladdin) at room temperature for 30 minutes. The resulting colored solution was centrifuged at 12 000*g* for 10 minutes at 4 °C to eliminate particulate impurities. 200 µL of supernatant were then transferred to 96-well plates. The absorbance of the solution was measured at 540 nm using a multifunctional microplate reader.

### 2.7 Immunofluorescence staining

In this study, Runx2 and BMP2 in cells on the scaffolds were immunolabeled to further evaluate the osteogenic activity of the material. Briefly, the cells fixed with 4% paraformaldehyde were blocked with 10% BSA and 1% Triton X-100. Then the cells were incubated with diluted primary antibodies against Runx2 and BMP2 at 4 °C overnight. After thoroughly washing with PBS, the cells were dyed with fluorescent secondary antibodies. And the DAPI staining was used to locate the nucleus. Finally, the cells were visualized under a fluorescence microscope (Nikon, E80i, Japan). And the mean fluorescence intensity was quantified using ImageJ software (NIH, USA).

### 2.8 Antioxidant assay

To assess the antioxidant capacity of the scaffolds, the reactive oxygen species (ROS) levels in cells co-cultured with the scaffolds were measured. First, 2 × 10^4^ cells were seeded onto glass slides coated with different material films and cultured for 24 hours. Hydrogen peroxide (H_2_O_2_) was then added to adjust its final concentration to 100 µmol L^−1^. After 12 hours of incubation, the cells were immersed in a 10 µM solution of 2′,7′-dichlorodihydrofluorescein diacetate (DCFH-DA) and incubated at 37 °C for 20 minutes. Finally, fluorescence images of the samples were captured using a fluorescence microscope (Nikon, E80i, Japan). The fluorescence intensity of the images was quantified using ImageJ software.

### 2.9 Animals and experimental protocol

All animal experiments were carried out according to the ARRIVE guidelines in accordance with National Research Council's Guide for the Care and Use of Laboratory Animals (eighth edition), and were performed under the approval of the Laboratory Animal Ethics Committee of Tongshi Biomedical (Changchun). Male SD rats (8 weeks old, weighing 200–240 g) were purchased from Liaoning Changsheng Biotechnology Co., Ltd (Benxi, China). Prior to the experiment, the rats were acclimated for 7 days under specific pathogen-free (SPF) conditions. Surgical procedures were conducted after anesthesia (3% sodium pentobarbital, 30 mg kg^−1^, i.p.). After shaving and disinfection (10% w/v povidone–iodine, 75% v/v ethanol), a *Φ* 3 × 3 mm bone defect on tibial bone was created with a trephine bur (800 rpm, Dentsply Sirona) under continuous saline irrigation (preserving dura). Then the defect was filled with the scaffold. Twenty-four defects were randomized into 6 groups (*n* = 4/group). After fed and cared separately for 8 weeks, the rats were sacrificed *via* overdose anesthesia (3% sodium pentobarbital, 150 mg kg^−1^, i.p.). The specimens were obtained and fixed in 4% paraformaldehyde. Micro-CT (ScanScan v1172, Bruker) was engaged to perform the bone regeneration. After scanning, the 3D images were reconstructed by Skyscan CTvox software (SkyScan 1172, Bruker). The representative indicators, including bone volume fraction (BV/TV), bone trabecular thickness (Tb. Th), bone trabecular number (Tb. N), and bone trabecular separation (Tb. SP), were calculated to evaluate the bone repair of the bone defect area by CTAn.

### 2.10 Histological evaluation

The harvested bone tissue specimens were decalcified in 15% ethylenediaminetetraacetic acid (EDTA, pH = 7.2) for 45 days for decalcification. After embedded in paraffin and sliced by electric slicer (Leica RM2016, US), the sections were dyed by Hematoxylin & Eosin (H&E) and Masson trichrome stain. Meanwhile, the immunofluorescent staining of BMP2 and TNF-α was performed on sections to evaluate osteogenesis and inflammatory. The scanning imaging system (NIKON DS-U3, Japan) were applied to capture the pictures of the stained section. In order to observe the type and maturity of collagen fibers in the defect area, sirius red was engaged to stain the tissue sections. A polarized light microscope (Zeiss, Axio Imager 2) was applied for observation and capture.

### 2.11 Biological safety evaluation of scaffolds

In order to evaluate the biological safety of the scaffolds implanted *in vivo*, the typical organs (heart, liver, spleen, lung, and kidney) of the sacrificed animals were collected. Fixed in 4% paraformaldehyde, the specimens were embedded in paraffin and sliced into tissue sections of 3 µm. The sections were stained by Hematoxylin & Eosin (H&E) and scanned by the scanning imaging system (NIKON DS-U3, Japan) to observe whether there are typical pathological features.

### 2.12 Statistical analysis

Data were presented as mean ± standard deviation (SD) of three independent tests unless special labeling. All the quantitative data were analyzed *via* Origin 8.0 software (Origin Lab Corporation, USA). The statistical analyses were performed by one-way analysis of variance (ANOVA). For two independent data comparisons, unpaired Student's *t*-test was used to determine statistical significance. For multiple comparisons of three or more sets of samples, one-way ANOVA with Turkey's comparison test was used. Comparisons with statistical differences (*p* < 0.05) were specifically flagged.

## 3 Result and discussion

### 3.1 Characterization of Res–Ce/HA nanoparticles

**Fig. 1:**
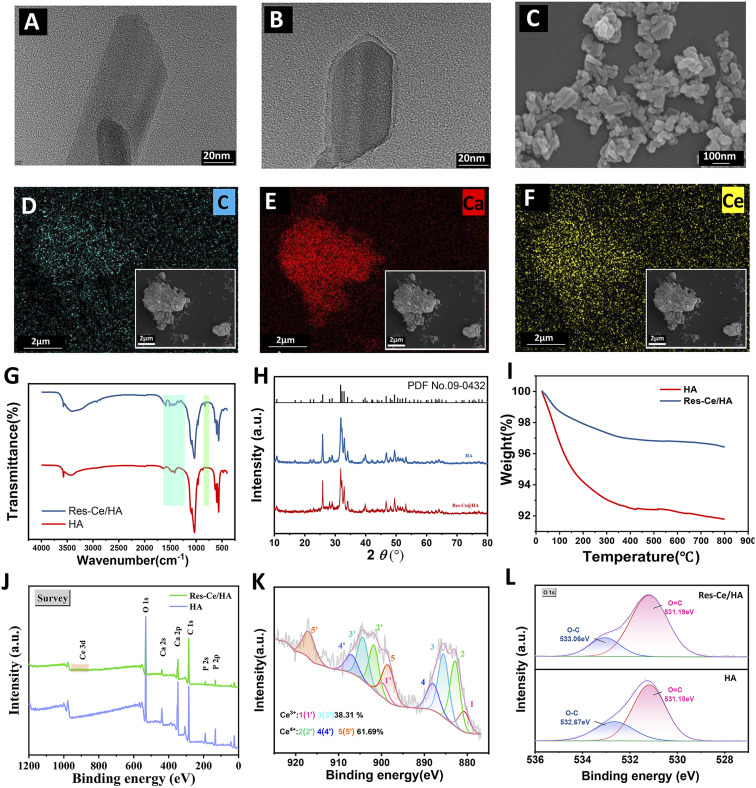
Characterization of Res–Ce/HA nanoparticles. (A and B) TEM photograph of HA and Res–Ce/HA nanoparticle. Bar = 20 nm. (C) SEM photograph of Res–Ce/HA nanoparticles. Bar = 100 nm. EDS mapping showing the element distributions of (D) C, (E) Ca and (F) Ce on Res–Ce/HA nanoparticles. Bar = 2 µm. (G) FT-IR and (H)XRD spectrum of HA and Res–Ce/HA nanoparticles. (I) Thermal weight loss curves of HA and Res–Ce/HA nanoparticles measured by TGA. (J) XPS spectrum of HA and Res–Ce/HA. (K) High resolution XPS spectra of Ce for Res–Ce/HA NPs. (L) High-resolution XPS spectra of the O 1s peak for HA and Res–Ce/HA.

The TEM photographs of single HA and Res–Ce/HA nanoparticles were shown in Fig. 1A and B. The nanoparticles exhibit a random short rod-like structure. The width of the particles was nearly 35 nm. In contrast to the HA nanoparticle, the surface of Res–Ce/HA nanoparticle was coated with a metal-phenolic network layer of 2–4 nm, which was composed of Res and Ce. The morphology shown in the SEM (Fig. 1C) indicated the agglomeration of particles. Furthermore, EDS mapping result (Fig. 1D–F) proved that the element distributions of C, Ca, and Ce in view were coincided on Res–Ce/HA nanoparticles. The Ca came from HA. And the C came from Res. This result indicated that the Res and Ce was mainly present on the HA nanoparticles. FT-IR analysis was employed to examine alterations in the distinctive functional groups of Res–Ce/HA nanoparticles. As depicted in Fig. 1G, the Res–Ce/HA nanoparticles exhibited several characteristic absorption peaks. A strong peak in the range of 1435 cm^−1^ indicates the O–C=O bond of Res.^35^ And the characteristic transmission bands of =CH_2_ (bending vibration) and –C–O (bending vibration) were assigned at 1362 and 1312 cm^−1^.^36^ Additionally, the transmission bands of Ce–O were assigned at 550 cm^−1^.^31^ The X-ray diffractograms (XRD) for the samples were shown in Fig. 1H. The nanoparticle is crystalline, and its main structure is hexagonal with the space group of *P*6_3_/*m* based on reference card no JCPDS 09-0432. The pattern of this powder has three remarkable peaks at 2*θ* values of 26°, 32°, and 35.6° related to (002), (211), and (202) planes, respectively.^37^ After modification with Res–Ce MPNs, the diffractions of HA were observed without any changing, which indicated that the modification with Res–Ce MPNs had no effect on the HA crystallinity. The XPS raw data were reasonably deconvoluted based on sample component. Full scan spectroscopy confirms that Res–Ce/HA nanoparticles are composed mainly of C, O, Ca, P, and Ce elements (Fig. 1J). The high resolution Ce 3d spectrum (Fig. 1K) exhibits series of sub peaks categorized as Ce 3d_3/2_ and Ce 3d_5/2_, substantiating the successful loading of Ce on Res–Ce/HA nanoparticle surface.^38^ Moreover, 38.31% of original Ce^4+^ was reduced into Ce^3+^, while 61.69% remain as Ce^4+^. The coexistence of Ce^4+^ and Ce^3+^ endows the material with inherent antioxidant potential. The high-resolution spectrum of O 1s in HA group coincided with the peaks at 531.18 eV (C=O) and 532.67 eV (C–O), while that in Res–Ce/HA group coincided with the peaks at 531.19 eV (C=O) and 533.06 eV (C–O) (Fig. 1L). These peaks in Res–Ce/HA group were enhanced, confirming the presence of Res. The thermal weight loss curves of HA and Res–Ce/HA nanoparticles measured by TGA were shown in Fig. 1I. The TGA profile of the Res–Ce/HA nanoparticles showed two weight loss steps from 20 to 400 °C (7.5%), and from 400 to 800 °C (0.5%). Compared to the Res–Ce/HA, the HA also showed two weight loss from 20 to 400 °C (3%) and from 400 to 800 °C (0.5%). Based on original weight, Res–Ce/HA lost 8% of weight, while the HA only lost 3.5% of its weight at 800 °C. This 5.5% difference between the weight loss could be attributed to the Res placed on the nanoparticle surface.

MPNs have found widespread applications in field of drug delivery.^27^ In the present study, in order to enhance the bioactivity utilization of Res and Ce, MPNs composed of Ce ions and Res was fabricated to modify the surface of HA nanoparticles. Owing to the abundant phenolic hydroxyl groups, the Res–Ce MPNs bond to HA nanoparticles through hydrogen bonding.^26^ According to the characterization results of Res–Ce/HA nanoparticles, the particle surface was successfully coated with the Res–Ce MPNs. During the reaction, 38.31% of original Ce^4+^ was reduced into Ce^3+^. In this situation, a favorable redox potential exists between Ce^4+^ and Ce^3+^. And its electronic structure confers excellent redox catalytic properties.^29^ Due to its outstanding nanozyme activity, the MPNs can rapidly respond to lower pH in an inflammatory microenvironment, achieving specific release of Res.^30^ This property endows the material with the potential to exert responsive functionality during the inflammatory phase of bone defect repair.

### 3.2 Characterization of composite scaffolds

**Fig. 2:**
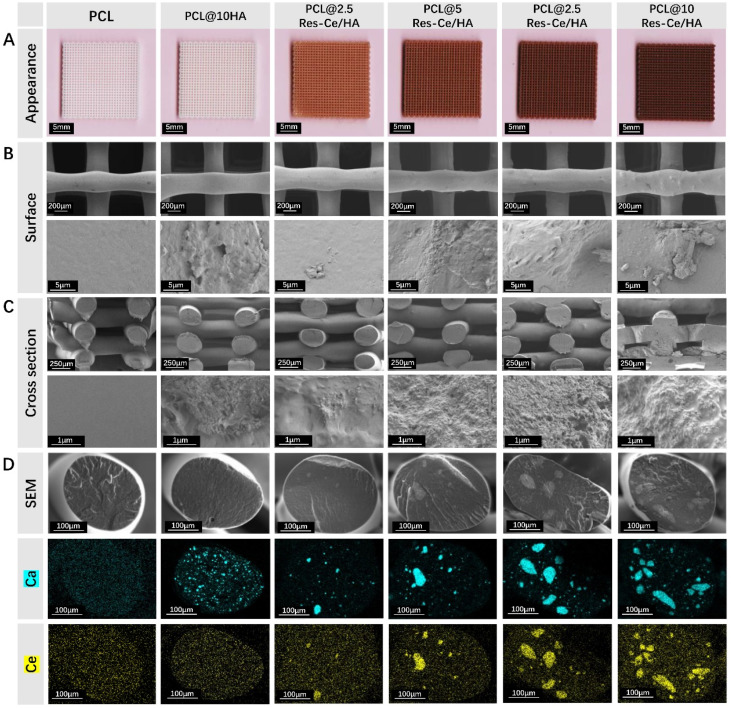
Microtopography of 3D printing composite scaffolds. (A) Appearance photos of scaffolds. Bar = 5 mm. (B) SEM photographs of scaffolds. Bar = 200 µm (low magnification) and 5 µm (high magnification). (C) SEM photographs of cross sections. Bar = 250 µm (low magnification) or 1 µm (high magnification). (D) EDS mapping showing the element distributions of Ca and Ce on cross sections. Bar = 100 µm.

The appearances of different scaffolds (PCL, PCL@10HA, PCL@2.5Res–Ce/HA, PCL@5Res–Ce/HA, PCL@7.5Res–Ce/HA and PCL@10Res–Ce/HA) were shown in Fig. 2A. While PCL and PCL@10HA groups maintained the characteristic white of native polymer, the PCL@Res–Ce/HA scaffolds were red in color due to the Ce^4+^ in the charge transfer state. Furthermore, the color darkened as the amount of Res–Ce/HA increased. SEM photos (Fig. 2B) confirmed preservation of the porous architecture across all modified scaffolds. Some of the added nanoparticles were exposed on the scaffold surface, rendering the microstructure of scaffolds rough. The greater the number of nanoparticles added, the rougher the scaffold surface became. Notably, the introduction of Res–Ce/HA nanoparticles did not significantly alter structural parameters of scaffolds. The SEM images of the cross sections (Fig. 2C) were consistent with those of the scaffold surfaces, with distinct discrete particles visible on the cross sections. EDS mapping of Ca and Ce was shown in Fig. 2D. The distributions of Res–Ce/HA nanoparticles in the cross-section were labeled *via* Ca (blue) and Ce (green). In contrast to the uniform distribution of HA in the PCL@HA scaffold, Res–Ce/HA nanoparticles were grumose in the 3D-printed scaffold. And the agglomeration became more pronounced as content of Res–Ce/HA nanoparticles increased. The reasons for the uneven dispersion of Res–Ce/HA nanoparticles in PCL could be attributed to several aspects. Due to abundant polar groups (such as hydroxyl and amino groups) on Res–Ce/HA MPNs, the significant difference in surface energy leads to poor interfacial adhesion between particles and PCL, which is weakly polar. In the other hand, Res–Ce/HA MPNs are prone to forming hydrogen bonds, coordination bonds, or π–π stacking between particles due to their structural characteristics. These strong intermolecular forces cause particles to self-aggregate, especially at high addition amounts, exacerbating dispersion unevenness.^27^

**Fig. 3:**
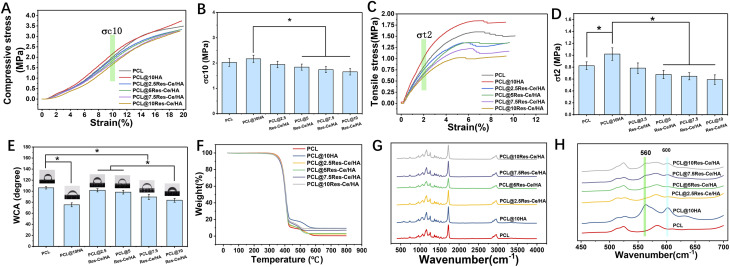
Physicochemical properties of 3D printing composite scaffolds. (A) Compressive stress–strain curves. (B) Compressive stress with a deformation of 10%. (C) Tensile stress–strain curves. (D) Tensile stress with a deformation of 2%. (E) Weight loss rate (%) of the scaffold after 21 days of degradation. (F) Thermal weight loss curves of different scaffolds measured by TGA. (G) FT-IR full-spectrum and (H) partial spectrum (from 400 cm^−1^ to 700 cm^−1^) of different scaffolds. *p < 0.05.

Fig. 3A presented the compressive stress–strain curves of the scaffolds. The curves of all the scaffolds showed a similar trend. In the initial segment of the curve (strain < 15%), a linear increase was observed, indicating that the scaffold behaved elastically under small deformations. As the strain reached 15% to 20%, a distinct yield plateau emerged, with a yield strength of approximately 3 MPa, signifying the onset of plastic deformation in the material. According to the result shown in Fig. 3B, compressive stress of PCL@10HA at 10% strain was significantly higher than those of PCL@5Res–Ce/HA, PCL@7.5Res–Ce/HA, and PCL@10Res–Ce/HA (*p* < 0.05). As shown in Fig. S1B, with the increase in the content of Res–Ce/HA in the scaffolds, the compressive modulus of the scaffolds exhibited a decreasing trend, and the compressive modulus of the PCL@10HA group was significantly higher than that of the PCL@10Res–Ce/HA group (*p* < 0.05). This result indicated that the incorporation of Res–Ce MPNs into the material reduced its compressive strength and compressive modulus. The tensile stress–strain curves of the scaffolds (Fig. 3C) showed marked differences between groups. All curves exhibited typical characteristics of plastic materials. In the initial stage (strain < 4%), there was a linear increase in each group, indicating that the materials were capable of elastic recovery under small deformations. When the strain reached 4% to 10%, the curves of all scaffolds showed a distinct yield plateau with a yield strength of from 1.0 MPa to 1.75 MPa, signifying the onset of plastic deformation. The tensile stress with a 2% strain of each group was shown in Fig. 3D. Compared with the PCL group, the tensile strength of PCL@10HA group obviously increased (*p* < 0.05). And it was also outstandingly higher than those of Res–Ce/HA containing groups (*p* < 0.05). As shown in Fig. S1D, the tensile modulus of the PCL@10HA group was significantly higher than that of the other groups. When the content of Res–Ce/HA in the scaffold was ≥ 5%, the tensile modulus of the scaffold was significantly lower than that of the PCL group (*p* < 0.05). This result demonstrated that the incorporation of HA effectively enhanced the tensile property of the PCL scaffold. Conversely, the introduction of Res–Ce MPNs decreased the tensile strength of the PCL@10HA scaffold. The incorporation of nHA particles to enhance the mechanical properties of polymer materials has been widely reported.^39,40^ As an inorganic rigid reinforcing phase, when uniformly dispersed in the polymer matrix, nHA can share external loads through interfacial interactions, reducing stress concentration in the polymer matrix and thereby improving the overall load-bearing capacity of the composite. However, the reinforcing effect of nHA depends on its dispersibility, interfacial compatibility with the matrix, and addition amount. The most significant reinforcement is achieved when nHA is uniformly dispersed with good interfacial bonding; agglomeration due to uneven dispersion or excessive addition may instead weaken performance due to stress concentration. From the SEM and EDS mapping results in Fig. 2, we observed that Res–Ce/HA aggregated within the PCL matrix, with this agglomeration becoming more pronounced as the addition amount increased. This led to uneven dispersion of the nanoparticles in PCL, thereby impairing the material's mechanical properties. This phenomenon is mainly attributed to the structural characteristics of resveratrol: resveratrol contains two benzene rings, each bearing a phenolic hydroxyl group. When the phenolic hydroxyl group on one benzene ring binds to HA, the phenolic hydroxyl group on the other end is exposed on the particle surface due to steric hindrance, and the exposed phenolic hydroxyl groups tend to aggregate *via* hydrogen bonding interactions. Such aggregation of Res–Ce/HA will lead to a decrease in the compressive strength and tensile stress of the scaffold.

After 21 days of degradation, the weight loss rate of each scaffold was calculated (Fig. 3E). The weight loss rates of the PCL, PCL/10HA, and PCL/2.5Res–Ce/HA scaffolds all remained below 5%. Notably, as the content of Res–Ce/HA nanoparticles increased, the weight loss resulting from material degradation increased significantly (*p* < 0.05). This finding indicates that the incorporation of Res–Ce/HA nanoparticles can significantly accelerate the degradation rate of PCL. PCL exhibits a slow degradation rate. Once implanted *in vivo*, it can undergo complete degradation within 1–2 years, which provides ample time for bone regeneration. However, this slow degradation also impedes new bone growth.^41,42^ EDS mapping results revealed the aggregation of Res–Ce/HA within the PCL matrix. This observation indicates that Res–Ce/HA possesses high hydrophilicity, which causes it to segregate from the hydrophobic PCL and aggregate in the matrix, forming hydrophilic cores. The degradation solution penetrates the PCL matrix through these hydrophilic cores, leading to an accelerated degradation rate. Thus, the incorporation of Res–Ce/HA enables regulation of the PCL degradation rate, thereby matching the scaffold's degradation rate with the pace of bone regeneration.

The thermal weight loss curves of scaffolds measured by TGA were shown in Fig. 3F. The TGA profile of the scaffolds showed three weight loss steps from 20 to 300 °C (2.5%), from 300 to 420 °C (0.5%), and from 420 to 800 °C. Compared to the Res–Ce/HA, the HA also showed two weight loss from 20 to 400 °C (3%) and from 400 to 800 °C (0.5%). The weight loss rate in first stage was little, indicating good thermal stability of the materials in range from 20 to 300 °C. The weight loss rate in second stage was approximately 75%, corresponding to the thermal decomposition of the PCL matrix. Based on original weight, the thermal weight loss of different materials varied in third stage. This difference between the weight loss could be attributed to the Res placed on the nanoparticle surface.

FT-IR full-spectrum and partial spectrum (from 400 cm^−1^ to 700 cm^−1^) of different scaffolds were shown in Fig. 3G and H. These data clearly revealed that the presence of the various vibrational modes corresponding to phosphates groups. As shown in this figure, the characteristics transmittance bands of the phosphate ion can be observed in the PCL containing nanoparticles. Within this range, the peaks at 560 and 600 cm^−1^ were assigned to PO_3_^−4^.^43^ However, with the introduction of Res–Ce MPNs, the peaks of phosphate has become less distinct.

### 3.3 Antibacterial property of composite scaffolds

**Fig. 4:**
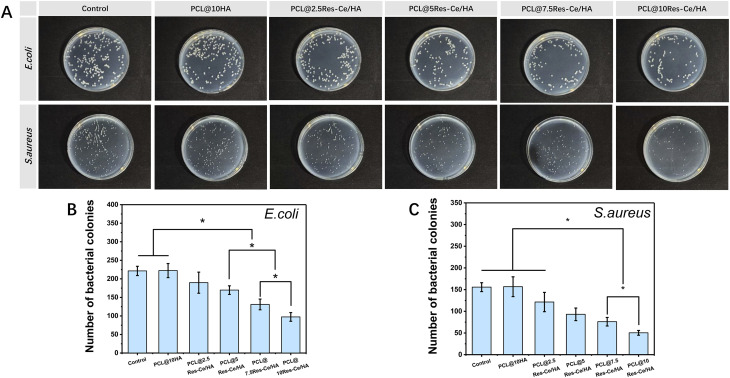
Antibacterial property of composite scaffolds. (A) Typical photos of the bacteria colonies formed on the solid LB agar plate. The statistical data on the material's antibacterial efficacy for (B) *Escherichia coli* and (C) *Staphylococcus aureus*. *p < 0.05.

Bacterial infection following scaffold implantation is a key factor contributing to excessive inflammatory responses during bone repair. In previous reports, both Res and Ce ions were believed to have antibacterial properties. Hwang' report has proven that Res antibacterial activity against *E. coli* is mediated by Z-ring formation inhibition *via* suppression of FtsZ expression.^44^ The antibacterial ability of Ce mainly relies on the reversible transformation between its chemical valence states (Ce^3+^ and Ce^4+^). Moreover, the synergy between Ce^3+^ and Ce^4+^ is more effective than a single valence state. It was reported that cerium oxide (CeO_2_) nanoparticles with an optimal ratio of the two valences (Ce^3+^ accounting for 20–40%) exhibit significantly enhanced antibacterial activity.^45^ Based on the results of material characterization in this study, during the reaction, 38.31% of original Ce^4+^ was reduced into Ce^3+^. So that the Res–Ce MPNs were expected to possess outstanding antibacterial property. According to the result (Fig. 4), As the content of Res–Ce/HA nanoparticles increased, the antibacterial performance of the scaffolds gradually improved. With the exception of the PCL@2.5Res–Ce/HA group, all other groups containing Res–Ce/HA nanoparticles exhibited significantly higher antibacterial activity than the PCL group and PCL@10HA group (*p* < 0.05). As shown in Fig. S2, the inhibition zone diameter of the scaffold increased with the increase in Res–Ce/HA content. These results were consistent with those in Fig. 4, further confirming that the higher the Res–Ce/HA content in the scaffold, the stronger its antibacterial activity. As shown in Table S1, the MICs of Res–Ce/HA nanoparticles against *E. coli* and *S. aureus* were 1.0 mg mL^−1^ and 0.8 mg mL^−1^, respectively. These results fully demonstrate that the incorporation of Res–Ce MPNs has significantly enhanced the antibacterial properties of the material. Owing to the characteristics of inorganic nanoparticles as antibacterial agents, including high antibacterial activity, long-lasting antibacterial performance, excellent biocompatibility, and favorable processability, the incorporation of antibacterial inorganic nanoparticles into matrices has become a universal strategy to endow bone repair composites with antibacterial capabilities.^46,47^

### 3.4 Osteogenic activity of composite scaffolds

**Fig. 5:**
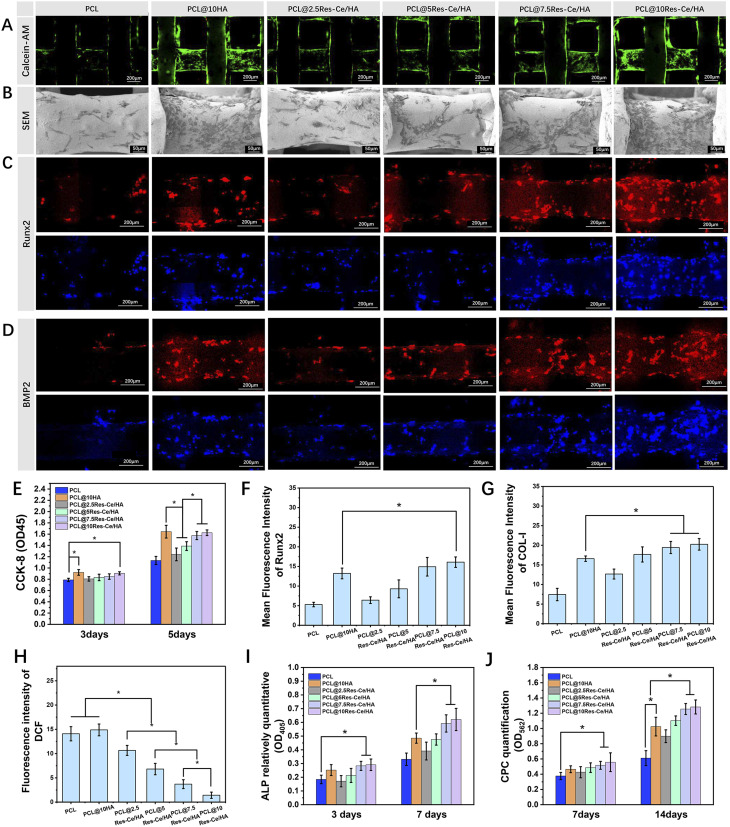
(A) The adhesion of MC3T3-E1 cells on the surface of each scaffold using calcein-AM staining (green fluorescence indicates living cells, while red fluorescence indicates dead cells); (B) SEM images of cell adhesion; (C and D) immunofluorescence staining images of intracellular Runx2 and BMP2 in cells on the scaffolds (red fluorescence indicates Runx2 and BMP2, while blue fluorescence indicates cell nuclei); (E) proliferation of MC3T3-E1 cells on the surface of scaffolds detected *via* CCK-8 assay; mean fluorescence intensity of (F) Runx2 and (G) BMP2 according to the immunofluorescence staining photos; (H) DCF fluorescent staining for intracellular ROS; (I) the relative ALP activity of MC3T3-E1 cells on scaffold in each group; (J) the quantitation results of ARS. *p < 0.05.

On the 3rd day of culture, the adhesion of MC3T3-E1 cells on the surface of each scaffold were assessed using Calcein AM/PI staining and SEM images. As shown in Fig. 5A, the green fluorescence staining indicates living cells on the scaffolds, while the red fluorescence indicates dead cells. Most of the cells on the scaffolds were living, with only a small number of dead cells present. As shown in Fig. 5B, the PCL@10HA and PCL@10Res–Ce/HA scaffolds exhibited the highest number of adherent cells on their surfaces. As the content of Res–Ce in the scaffolds decreased, the number of adherent cells on the scaffold surfaces also decreased accordingly. The PCL group showed the minimum number of adherent cells. As shown in Fig. S3, the number of adherent cells on the scaffolds of different groups exhibited a consistent trend with that in Fig. 5A and B. The number of adherent cells on the scaffolds of the PCL@10HA group and PCL@10Res–Ce/HA group was significantly higher than that of the other groups. Cell proliferation at different time points was shown in Fig. 5E. The trend of cell proliferation in each group was consistent with the cell adhesion results. On day 3, compared with the PCL group, cell proliferation in the PCL@10HA and PCL@10Res–Ce/HA groups was significantly accelerated (*p* < 0.05). On day 5, cell proliferation in the PCL@10HA, PCL@7.5Res–Ce/HA, and PCL@10Res–Ce/HA groups was significantly higher than that in the other groups (*p* < 0.05). These results fully indicated that both HA and Res–Ce/HA nanoparticles in the scaffolds could promote the adhesion and proliferation of MC3T3-E1 cells. During the implantation, osteoblasts recruited from the surrounding tissue must first adhere to the scaffold surface and colonize to proceed the subsequent bone repair process.^48^ The results of this study indicated that the PCL@Res–Ce/HA scaffold could effectively promote the adhesion and proliferation of osteoblasts on its surface, which might be associated with the scaffold's rough surface. This conclusion provides a significant basis for the application of PCL@Res–Ce/HA scaffolds in bone repair.

The intracellular Runx2 and BMP2 on the scaffold were labeled by immunofluorescence staining (Fig. 5C and D). The stained Runx2 and BMP2 in PCL@10HA, PCL@5Res–Ce/HA, PCL@7.5Res–Ce/HA, and PCL@10Res–Ce/HA groups were much more obvious in comparison with that in PCL group. And the red dyeing of Runx2 and BMP2 in the PCL@2.5Res–Ce/HA group was not more remarkable than in the PCL group. The immunofluorescence staining of Runx2 and BMP2 was quantified and shown in Fig. 5F and G. The statistical results were consistent with the immunofluorescence staining images. Compared with the PCL@10HA group, the PCL@10Res–Ce/HA group showed accelerated Runx2 expression (*p* < 0.05). Regarding BMP2 expression, both the PCL@7.5Res–Ce/HA and PCL@10Res–Ce/HA groups exhibited significantly higher levels than the PCL@10HA group (*p* < 0.05). However, in terms of promoting BMP2 expression, the PCL@2.5Res–Ce/HA group had an equivalent effect to the PCL@10HA group. Numerous *in vitro* and *in vivo* studies have underscored bone–protective properties of Res, highlighting its role as a stimulator of osteoblast proliferation and an antagonist of osteoclast differentiation.^19^ Recent studies on the mechanism of Res in bone repair have been gradually lucubrated. Res can alleviate the inhibition of osteogenic differentiation of bone marrow mesenchymal stem cells induced by tumor necrosis factor-α (TNF-α), thereby delaying the progression of osteoporosis. The enhancement of the Wnt signaling pathway, activation of sirtuin 1 (Sirt1), and acetylation of runt-related transcription factor 2 (Runx2) have been identified as potential mechanisms through which Res exerts these effects.^20^ Furthermore, Res was also proved to activate the Src kinase-dependent estrogen receptor (ER) to stimulate osteoblasts to produce bone morphogenetic protein 2 (BMP2), while also increasing the serum concentration of BMP-2 *in vivo*.^21^ Moreover, Ce can also upregulate the expression of osteogenesis-related genes such as Runx2 and BMP2.^49,50^ The results of this study indicated that Res–Ce MPNs retained the osteogenic properties of Res and Ce. Through the modification of Res–Ce MPNs, the osteogenic activity of HA nanoparticles had also been enhanced.

In Fig. S4, intracellular reactive oxygen species (ROS) are highlighted in green by DCFH-DA. The PCL and PCL/10HA groups showed the highest fluorescence intensity, whereas the fluorescence intensity decreased gradually as the content of Res–Ce/HA increased. The fluorescence intensity of DCF was detected to quantify the intracellular ROS levels (Fig. 5H). According to the results, the intracellular ROS level in the PCL@10HA group was equal to that in the PCL group, yet significantly higher than that in the Res–Ce/HA containing groups (*p* < 0.05). Notably, as the content of Res–Ce/HA nanoparticles increased, the ROS level decreased significantly (*p* < 0.05). Among all groups, the PCL@10Res–Ce/HA group exhibited the lowest intracellular ROS level, which was significantly lower than that in the other groups (*p* < 0.05). Bone defect areas are often accompanied by the accumulation of superoxide and ROS, which can further induce cell apoptosis and excessive inflammation, and delay the repair process. Res has been confirmed to possess significant antioxidant activity.^13^ Additionally, the coexistence of Ce^3+^ and Ce^4+^ in the material significantly enhances the nanozyme activity of Res–Ce MPNs, thereby exerting an antioxidant effect.^29^ The findings of this study indicate that scaffolds containing Res–Ce/HA nanoparticles can effectively scavenge intracellular ROS. This function helps alleviate the inflammatory response at bone injury sites and promotes tissue repair.

The results of relative ALP activity were shown in Fig. 5I. On day 3, only the PCL@7.5Res–Ce/HA and PCL@10Res–Ce/HA groups exhibited higher relative ALP activity than the PCL group (*p* < 0.05). Although the PCL@10HA group showed a higher level than the PCL group, the difference was not significant. On day 7, the PCL@10HA group had significantly higher ALP activity than the PCL group (*p* < 0.05). Furthermore, the relative ALP activity of the PCL@7.5Res–Ce/HA and PCL@10Res–Ce/HA groups was significantly higher than that of the PCL@10HA group (*p* < 0.05). Fig. 5J illustrates the CPC quantitation results on days 7 and 14. At the 7-day time point, the PCL@7.5Res–Ce/HA and PCL@10Res–Ce/HA groups were the only ones that demonstrated significantly elevated CPC quantitation values compared to the PCL group (*p* < 0.05). By day 14, the PCL@10HA group exhibited a significant increase in CPC quantitation relative to the PCL group (*p* < 0.05). Moreover, both the PCL@7.5Res–Ce/HA and PCL@10Res–Ce/HA groups showed significantly higher CPC quantitation values than the PCL@10HA group (*p* < 0.05).

The ability to induce osteogenic differentiation is critical for evaluating the biological activity of bone repair materials. ALP activity level serves as a marker for the early stage of cellular osteogenic differentiation, while cellular calcium deposition is recognized as a late-stage indicator of osteoblastic differentiation.^51,52^ The results of this study confirmed the osteogenic activity of HA nanoparticles. However, the HA nanoparticles within the scaffold did not exhibit remarkable osteogenic activity in the early stage. In contrast, the Res–Ce/HA nanoparticles demonstrated significantly prominent osteogenic activity in both the early and late stages. These findings further provide a crucial basis for the application of PCL@Res–Ce/HA scaffolds in bone repair.

### 3.5 Assessment of bone formation using micro-CT

**Fig. 6:**
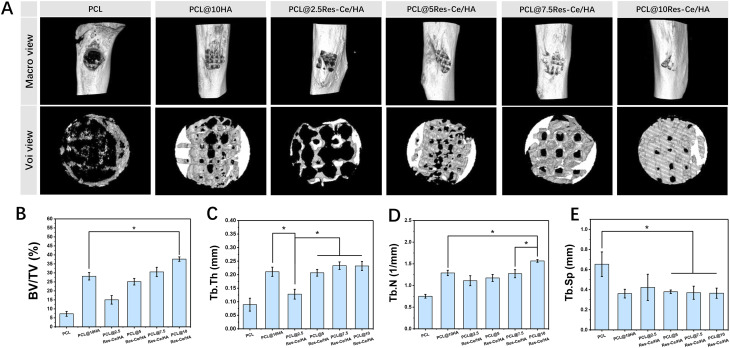
Evaluation of scaffolds on the rat tibial defect repair. (A) 3D model reconstructions of tibial injury specimens and new bone in the defect area based on micro-CT data. Quantitatively analysis of (B) bone mass (BV/TV), (C) trabecular thickness (Tb.Th), (D) trabecular number (Tb.N) and (E) trabecular separation (Tb.Sp) at the defect site, *n* = 3 *p < 0.05.

Micro-CT was used to scan the tibial injury specimens and reconstruct 3D models of new bone in the defect area. The results of 3D tissue reconstruction were shown in Fig. 6A. All groups exhibited improved coverage of new tissue over the defect. New bone grew from the edges toward the center along the scaffold structure. Compared with the PCL group, the PCL/10HA group showed superior new bone regeneration. Among the Res–Ce/HA-containing groups, the coverage area of new tissue in the defect gradually increased with the rising content of Res–Ce/HA nanoparticles, with the PCL@10Res–Ce/HA group achieving almost complete coverage of the defect surface.

**Fig. 7:**
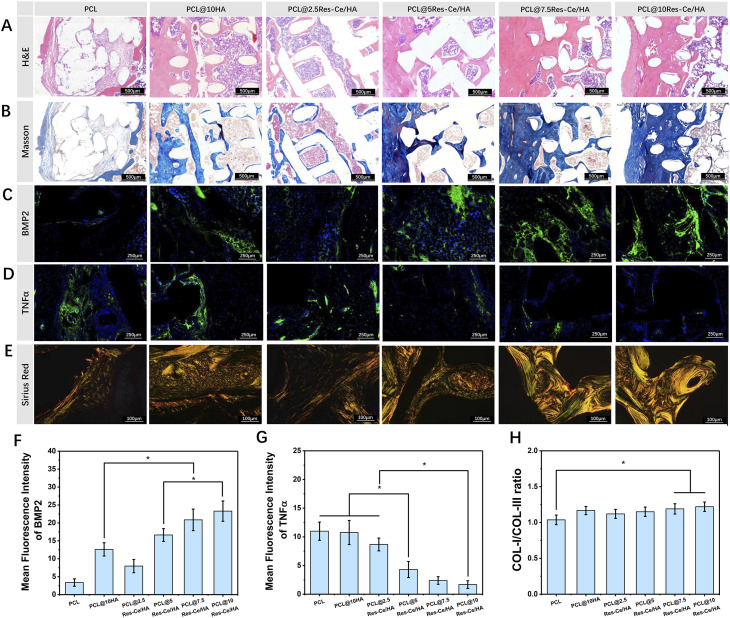
Histological evaluation of rat tibial bone defect repair. (A) H&E staining images; (B) Masson trichrome staining images; (C) immunofluorescence staining images of BMP2 and (D) TNF-α (where green fluorescence indicates BMP2 and TNF-α, and blue fluorescence indicates cell nuclei); (E) sirius red staining images. (F) Relative fluorescence intensity of BMP2 based on immunofluorescence images; (G) Relative fluorescence intensity of TNF-α based on immunofluorescence images. (H) Ratio of COL-I/COL-III based on sirius red staining. Scale bars are indicated in the respective images. N = 3, *p < 0.05.

To quantify the orbital bone repair effect of each scaffold, CTAn was used to analyze the bone mass (BV/TV), trabecular thickness (Tb.Th), trabecular number (Tb.N), and trabecular separation (Tb.Sp) at the injured site. The results are shown in Fig. 7B–E. BV/TV represents the content of new bone tissue per unit volume and serves as a key indicator for evaluating bone repair. The results revealed that as the content of PCL@Res–Ce/HA nanoparticles increased, the BV/TV at the defect site increased accordingly. Among all groups, the BV/TV value of the PCL@10Res–Ce/HA group was significantly higher than that of the PCL/10HA group (*p* < 0.05). Bone trabeculae refer to the projections of cortical bone extending into cancellous bone. Their primary functions include connecting bone tissue components, preserving bone stability and the integrity of its overall structure, offering internal support to the bone, and contributing to bone growth and development processes. The thickness, quantity, and spacing of bone trabeculae are mutually correlated metrics, and together they serve as a reflection of the bone's internal structural characteristics. In this study, the Tb.Th of the PCL@10Res–Ce/HA group was significantly greater than that of the PCL group (*p* < 0.05). Meanwhile, its Tb.N was also significantly higher than those of both the PCL group and the PCL@10HA group (*p* < 0.05). Although the Tb.Th of the PCL@10HA group was lower than that of the PCL@10Res–Ce/HA group, there was no statistically significant difference between the two groups. Additionally, the Tb.Sp of the PCL group was significantly higher than that of all other groups except the PCL@2.5Res–Ce/HA group (*p* < 0.05). These findings fully demonstrated that the PCL@10Res–Ce/HA group exerted an excellent promotional effect on bone repair.

### 3.6 Histological assay

In this study, H&E staining was employed to observe the tissue morphology of the fracture defect area at 8 weeks post-repair, with the results presented in Fig. 7A. Scaffolds in all groups were encapsulated by connective tissue, and tissue extensions had grown into the interior of the scaffolds through their pores. The PCL scaffold was primarily surrounded and infiltrated by loose fibrous connective tissue, with a large number of inflammatory cells visible. In contrast, the connective tissue surrounding and inside the composite scaffolds containing HA or Res–Ce/HA nanoparticles was denser. These composite scaffolds also contained abundant new bone tissue and only a small number of inflammatory cells. Among all groups, the PCL@10Res–Ce/HA group exhibited the densest new tissue formation. Fig. 7B presents the results of Masson staining. In these images, blue regions correspond to newly formed collagen-rich bone tissue, whereas red regions indicate mature bone tissue. The findings revealed that the PCL scaffold was primarily surrounded by loose collagen fibers, with only a few scattered red regions observable within the newly formed bone tissue. As the content of Res–Ce/HA nanoparticles increased, the density of new tissue both around and inside the scaffold gradually rose. Additionally, more red-stained areas were detected within the new bone tissue—an observation that suggests the progressive maturation of the new bone. Among all groups, the PCL@10Res–Ce/HA group exhibited the greatest number and largest size of red-stained regions, which indicates that the new bone in this group had achieved the highest level of maturity. Collectively, these results demonstrate that the incorporation of Res–Ce/HA nanoparticles effectively facilitated the formation and maturation of new bone.

BMP2 is a growth factor which plays a key role in osteoblast differentiation and bone regeneration.^53^ In this study, immunofluorescence staining was performed to detect the content of BMP2 in the fracture defect, with representative images displayed in Fig. 7C. As shown in the staining results, the PCL group exhibited the lowest level of BMP2. In contrast, the tissue-level BMP2 expression gradually increased with the elevated content of Res–Ce/HA. Notably, in the groups with HA or Res–Ce/HA nanoparticles, BMP2 was mainly enriched in the tissue regions adjacent to the scaffolds. Quantitative analysis of relative fluorescence intensity, derived from the immunofluorescence images, is presented in Fig. 7F. Among all experimental groups, the PCL@10Res–Ce/HA group showed the highest relative fluorescence intensity of BMP2, which was significantly greater than that in the PCL/HA, PCL@2.5Res–Ce/HA, and PCL@5Res–Ce/HA groups (*p* < 0.05). These findings fully confirm that Res–Ce/HA nanoparticles in the composite scaffolds can significantly upregulate BMP2 expression in local tissues, thereby further facilitating bone regeneration and repair. Moreover, this promotional effect exhibits a positive correlation with the content of Res–Ce MPNs in the scaffold. This was consistent with the results of the *in vivo* experiments.

Res was proven to offer the properties of antioxidant^13^ and anti-inflammatory.^14^ Additionally, it exhibits significant antibacterial activity against various pathogens.^18^ Ce and its ions are also believed to possess antibacterial and antioxidant properties.^29,45^ Base on the above research, the anti-inflammatory property represented another anticipated advantage of Res–Ce MPNs. To evaluate this property of the composite scaffolds, immunofluorescent staining was used to detect TNF-α expression at the defect site. As illustrated in Fig. 7D, the green-labeled TNF-α in the Res–Ce/HA-containing groups was significantly less abundant than that in the PCL and PCL/HA groups. Furthermore, as the content of Res–Ce/HA increased, the expression level of TNF-α in the local tissue gradually decreased. The quantitative analysis results of TNF-α immunofluorescence are presented in Fig. 7F. The PCL@10Res–Ce/HA group exhibited the lowest relative fluorescence intensity of TNF-α, which was significantly lower than that in all other groups except the PCL@7.5Res–Ce/HA group (*p* < 0.05). Although the expression level of TNF-α in the PCL@7.5Res–Ce/HA group was slightly higher than that in the PCL@10Res–Ce/HA group, the difference between the two groups was not statistically significant. These findings confirm that Res–Ce MPNs exert an inhibitory effect on inflammation during the bone repair process.

Sirius red staining was further employed to assess the arrangement, distribution, and maturity of collagen fibers in regenerated bone tissue, with the results presented in Fig. 7E. In the staining images, red and yellow fibers corresponded to type I collagen—an abundant component in bone tissue—while green fibers represented type III collagen. Type III collagen is immature and unstable, and it is primarily found in cartilage tissue. The results indicated that collagen fibers around and within the PCL scaffolds were loosely and randomly arranged. In contrast, collagen fibers surrounding and inside the scaffolds containing HA or Res–Ce/HA nanoparticles were thicker and densely packed. As the content of Res–Ce/HA nanoparticles increased, both the thickness and density of collagen fibers increased accordingly. Furthermore, the proportion of red and yellow type I collagen also significantly rose with increasing Res–Ce/HA nanoparticle content (*p* < 0.05), which was validated by quantitative analysis of the stained images (Fig. 7H).

### 3.7 Biological safety of composite scaffolds

**Fig. 8:**
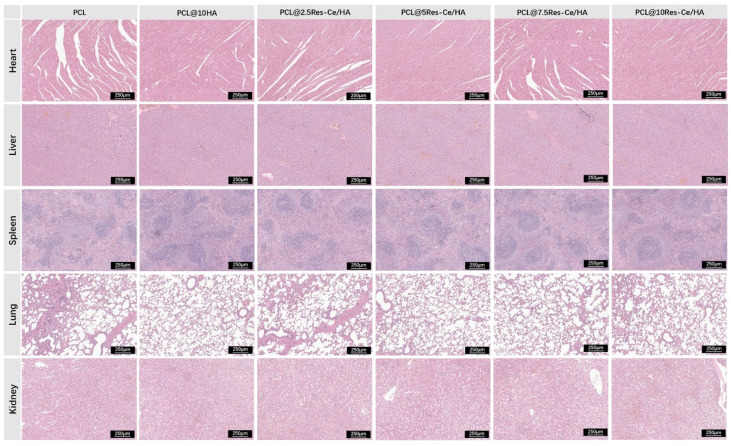
H&E staining of major organs (heart, liver, spleen, lung, and kidney) to assess potential organ-specific toxicity of scaffolds. Bar = 200 µm.

Biological safety assessment is still essential for implantable materials, as degradation byproducts of scaffolds may trigger biotoxicity through hematogenous dissemination. To evaluate potential organ-specific toxicity, comprehensive histological analyses were conducted on major organs (heart, liver, spleen, lung, and kidney) 8 weeks post-implantation. Histological observations *via* H&E staining (Fig. 8) showed that tissue integrity was well preserved across all experimental groups. Tissue cells were arranged tightly, and no obvious hemorrhage, inflammatory cell infiltration, or cellular degeneration and necrosis were detected in any of the examined tissues. The lack of histopathological abnormalities in vital organ systems indicates excellent systemic biocompatibility, which verifies the clinical applicability of PCL@Res–Ce/HA composite scaffolds for bone reconstruction.

## 4 Conclusions

In this study, Res–Ce MPNs-modified HA nanoparticles (Res–Ce/HA nanoparticles) were constructed as the inorganic phase and blended with PCL to fabricate multifunctional 3D-printed bone repair scaffolds, which endows the constructs with synergistic antibacterial, anti-inflammatory, and osteogenic activities; rigorous *in vitro* and *in vivo* assays combined with biosafety evaluations fully validate the clinical potential of these scaffolds. Specifically, the composite scaffolds incorporating Res–Ce/HA nanoparticles exhibited enhanced antibacterial activity, and *in vitro* evaluations confirmed that the PCL@10Res–Ce/HA scaffold possessed significant advantages in cytocompatibility and osteogenic potential. Furthermore, rat tibial defect repair experiments demonstrated that the 3D-printed PCL@10Res–Ce/HA scaffold remarkably promoted osteogenesis at the defect site through three synergistic mechanisms: accelerating new bone formation and maturation, guiding tissue ingrowth into the scaffold's internal structure, and upregulating BMP-2 expression, while the Res–Ce MPNs in the scaffold also inhibited excessive inflammation to facilitate bone regeneration. Importantly, comprehensive biosafety assessments validated the clinical feasibility of the PCL@10Res–Ce/HA scaffold, and collectively, these findings indicate that the PCL@Res–Ce/HA scaffold with optimized composition integrates anti-inflammatory, immunomodulatory, and bone defect repair functions, rendering it a promising candidate material for bone defect repair. However, the uneven dispersion of Res–Ce/HA nanoparticles compromises mechanical performance, while the long-term degradation behavior and scaffold-tissue crosstalk remain elusive, and future work should focus on optimizing nanoparticle dispersion through surface modification, extending *in vivo* follow-up durations, employing large-animal models to recapitulate clinical settings, and elucidating the molecular mechanisms underlying Res–Ce MPN-mediated bone regeneration to facilitate clinical translation.

## Author contributions

Dezhou Wang: writing – original draft, methodology, investigation, data curation. Min Guo: review & editing, formal analysis, conceptualization. Yuqi Gao: methodology, investigation. Shengrui Gao: investigation, data curation. Wanzhong Yin: review & editing, visualization. Wenzhi Song: writing – review & editing, conceptualization, supervision.

## Conflicts of interest

There are no conflicts to declare.

## Abbreviations

ALP: Alkaline phosphatase
ANOVA: One-way analysis of variance
ARS: Alizarin red S
BMP2: Bone morphogenetic protein 2
BMP9: Bone morphogenetic protein 9
BSA: Bovine serum albumin
BV/TV: Bone volume fraction
CAT: Catalase
CCK-8: Cell counting kit-8
CFU: Colony-forming units
CPC: Cetylpyridinium chloride
COL-I: Collagen type I
COL-III: Collagen type III
DCF: 2′,7′-Dichlorofluorescein
DCFH-DA: 2′,7′-Dichlorodihydrofluorescein diacetate
DAPI: 4′,6-Diamidino-2-phenylindole
EDTA: Ethylenediaminetetraacetic acid
*E. coli*
: *Escherichia coli*
EDS: Energy dispersive spectrometer
ER: Estrogen receptor
EGCG: Epigallocatechin gallate
FBS: Fetal bovine serum
FDM: Fused deposition modeling
FT-IR: Fourier transform infrared spectroscopy
GNP: Gelatin nanoparticle
H&E: Hematoxylin & eosin
H_2_O_2_: Hydrogen peroxide
HA: Hydroxyapatite
IL-1β: Interleukin-1β
MIC: Minimum inhibitory concentration
MRSA: Methicillin resistant *Staphylococcus aureus*
MPNs: Metal-phenolic networks
NPs: Nanoparticles
nHA: Nano-hydroxyapatite
OXD: Oxidase
PCL: Polycaprolactone
PMSF: Phenylmethanesulfonyl fluoride
POD: Peroxidase
pNPP: *p*-Nitrophenyl phosphate
Res: Resveratrol
ROS: Reactive oxygen species
RNS: Reactive nitrogen species
Runx2: Runt-related transcription factor 2
SEM: Scanning electron microscopy
Sirt1: Sirtuin 1
SOD: Superoxide dismutase
SPF: Specific pathogen-free
TEM: Transmission electron microscope
Tb.N: Bone trabecular number
Tb.Sp: Bone trabecular separation
Tb.Th: Bone trabecular thickness
TNF-α: Tumor necrosis factor-α
TGA: Thermogravimetric analysis
VEGF: Vascular endothelial growth factor
XPS: X-ray photoelectron spectroscopy
XRD: X-ray diffraction

## Supplementary Material

RA-016-D5RA10060G-s001

## Data Availability

All relevant data supporting the findings of this research are available from the corresponding author upon reasonable request. Supplementary information (SI): supplementary figures and tables that support the results presented in the manuscript. See DOI: https://doi.org/10.1039/d5ra10060g.
