# Supplementary material for: Synthesis and evaluation of resveratrol–cerium modified hydroxyapatite for enhanced bone repair scaffolds
Source: RSC Adv. 2026 Feb 23;16(12):10565–81. doi: 10.1039/d5ra10060g (PMC12926797; doi:10.1039/d5ra10060g)
Supplement: RA-016-D5RA10060G-s001 [file RA-016-D5RA10060G-s001.pdf]

Supplementary Information (SI) for RSC Advances.
This journal is © The Royal Society of Chemistry 2026

Supplementary Materials

Synthesis and Evaluation of Resveratrol-Cerium Modified Hydroxyapatite for Enhanced Bone Repair Scaffolds

Dezhou Wang[a], Min Guo[b], Yuqi Gao[a], Shengrui Gao[c], Wanzhong Yin[c*], Wenzhi Song[a*]

[a] *Stomatology Department, China-Japan Union Hospital of Jilin University, Changchun, 130033, PR China*

[b] *State Key Laboratory of Polymer Science and Technology, Changchun Institute of Applied Chemistry, Chinese Academy of Sciences, Changchun, 130022, PR China*

[c] *Department of Otorhinolaryngology, The First Hospital of Jilin University, 1 Xinmin Street, Changchun 130021, PR China*

*Correspondence should be addressed to Wenzhi Song (songwz@jlu.edu.cn) and Wanzhong Yin (yinwz@jlu.edu.cn)

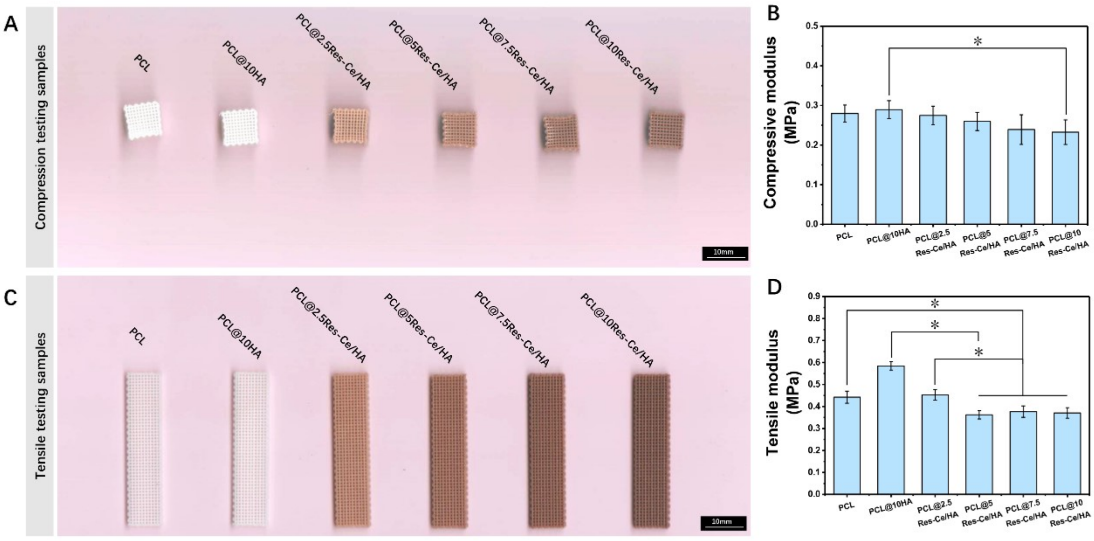

Fig. S1 Appearance images of the samples used for (A) the compression (10×10×10mm) and (C) tensile (10×40×2mm) mechanical tests of the scaffold; (B) Tensile moduli and (D) compressive moduli of the samples, scale bar = 10 mm (n=3, *p < 0.05).

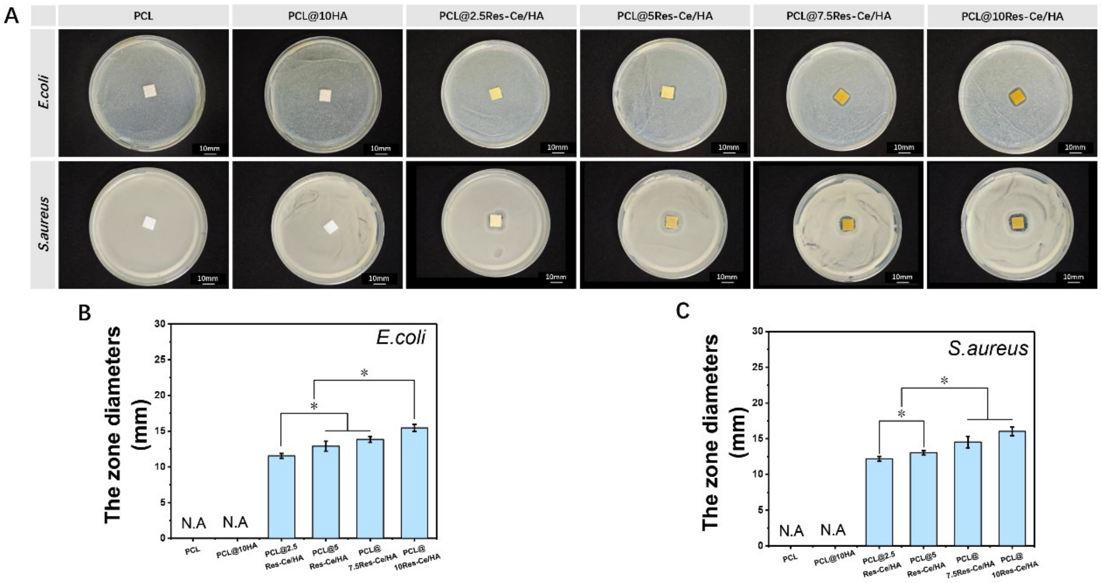

Fig. S2 (A) Inhibition zone images of scaffolds in different groups against *E. coli* and *S. aureus*. Inhibition zone diameters against (B) *E. coli* and (C) *S. aureus* (n=3, *p < 0.05).

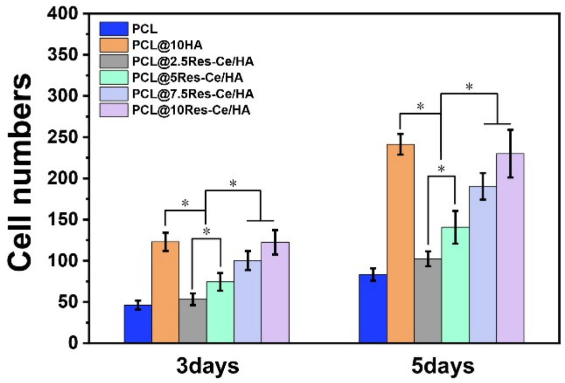

Fig. S3 The number of cells on the scaffolds from different groups after 3 and 5 days of culture, , n=3 *$p < 0.05$.

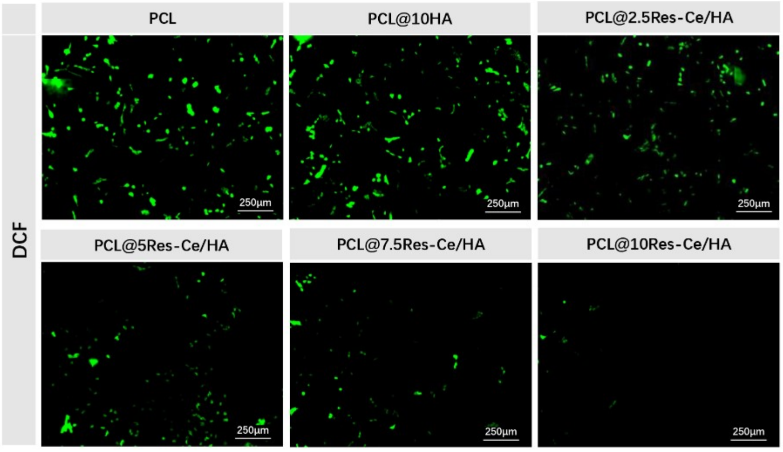

Fig. S4 Intracellular ROS detection (Scale bar = 250 μm).

Table S1. MIC Values of Res-Ce/HA Nanoparticles against *E. coli* and *S. aureus*

	MIC (mg/mL)
E.coli (Gram-)	1.2
S. aureus(Gram+)	1.0
